# *N*-[(1,3-Benzodioxol-5-yl)meth­yl]benzene­sulfonamide: an analogue of capsaicin

**DOI:** 10.1107/S1600536813028481

**Published:** 2013-10-23

**Authors:** Stella H. Maganhi, Maurício T. Tavares, Mariana C. F. C. B. Damião, Roberto Parise Filho

**Affiliations:** a Department of Physics, Universidade Federal de São Carlos, 13565-905 São Carlos, SP, Brazil; b Department of Pharmacy, Universidade de São Paulo, 05508-000 São Paulo, SP, Brazil

## Abstract

The title compound, C_14_H_13_NO_4_S, an analogue of capsaicin, differs from the latter by having a 1,3-benzodioxole ring rather than a 2-meth­oxy­phenol moiety, and having a benzene­sulfonamide group instead of an aliphatic amide chain. The five-membered ring is in an envelope conformation with the methyl­ene C atom lying 0.221 (6) Å out of the plane formed by the other four atoms. The dihedral angle between the phenyl ring and the mean plane of the 1,3-benzodioxole fused-ring system is 84.65 (4)°. In the crystal, mol­ecules aggregate into supra­molecular layers in the *ac* plane through C—H⋯O, N—H⋯O and C—H⋯π inter­actions.

## Related literature

For background and the biological activity of capsaicin, see: Lee *et al.* (2011 ▶); Malagarie-Cazenave *et al.* (2011 ▶). For the synthesis and cytoxicity of the title compound, see: De Sá-Junior *et al.* (2013 ▶). For ring conformational analysis, see: Cremer & Pople (1975 ▶).
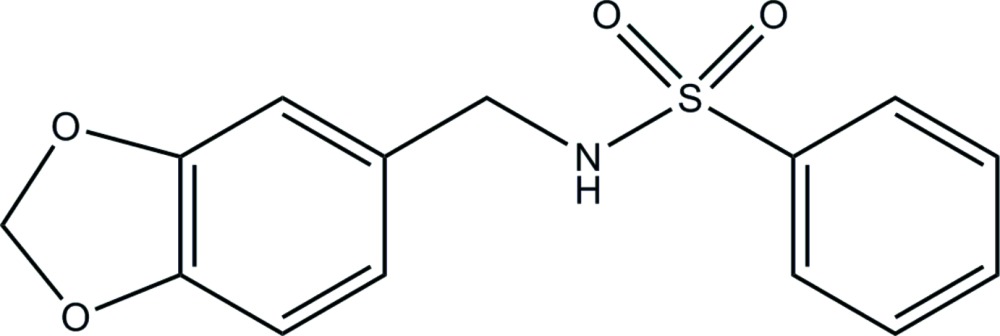


## Experimental

### 

#### Crystal data

C_14_H_13_NO_4_S
*M* *_r_* = 291.32
Orthorhombic, 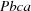

*a* = 18.0158 (4) Å
*b* = 5.9346 (1) Å
*c* = 25.5480 (8) Å
*V* = 2731.51 (11) Å^3^
*Z* = 8
Mo *K*α radiation
μ = 0.25 mm^−1^
*T* = 290 K
0.25 × 0.22 × 0.20 mm

#### Data collection

Nonius KappaCCD diffractometer with Bruker APEXII CCD areadetector
Absorption correction: multi-scan (*SADABS*; Sheldrick, 1996 ▶) *T*_min_ = 0.930, *T*_max_ = 0.948
2652 measured reflections
2652 independent reflections
1860 reflections with *I* > 2σ(*I*)

#### Refinement

*R*[*F* ^2^ > 2σ(*F* ^2^)] = 0.051
*wR*(*F* ^2^) = 0.149
*S* = 1.04
2652 reflections
181 parameters
H-atom parameters constrained
Δρ_max_ = 0.26 e Å^−3^
Δρ_min_ = −0.29 e Å^−3^

### 

Data collection: *COLLECT* (Nonius, 1999 ▶); cell refinement: *SCALEPACK* (Otwinowski & Minor, 1997 ▶); data reduction: *DENZO* (Otwinowski & Minor, 1997 ▶) and *SCALEPACK*; program(s) used to solve structure: *SIR97* (Altomare *et al.*, 1999 ▶); program(s) used to refine structure: *SHELXL97* (Sheldrick, 2008 ▶); molecular graphics: *ORTEP-3 for Windows* (Farrugia, 2012 ▶); software used to prepare material for publication: MarvinSketch (ChemAxon, 2010 ▶) and *publCIF* (Westrip, 2010 ▶).

## Supplementary Material

Crystal structure: contains datablock(s) global, I. DOI: 10.1107/S1600536813028481/tk5268sup1.cif

Structure factors: contains datablock(s) I. DOI: 10.1107/S1600536813028481/tk5268Isup2.hkl

Supplementary material file. DOI: 10.1107/S1600536813028481/tk5268Isup3.cml

Additional supplementary materials:  crystallographic information; 3D view; checkCIF report

## Figures and Tables

**Table 1:** Hydrogen-bond geometry (Å, °) *Cg*1 and *Cg*2 are the centroids of the C2–C7 and C9–C14 rings, respectively.

*D*—H⋯*A*	*D*—H	H⋯*A*	*D*⋯*A*	*D*—H⋯*A*
N1—H1*N*⋯O2^i^	0.99	2.00	2.945 (3)	157
C14—H14⋯O1^ii^	0.93	2.59	3.486 (3)	161
C10—H10⋯*Cg*1^iii^	0.93	2.74	3.563 (3)	147
C8—H8*B*⋯*Cg*2^iv^	0.97	2.82	3.511 (3)	129

Symmetry codes: (i) 
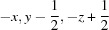
; (ii) 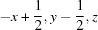; (iii) 
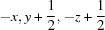
; (iv) 
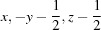
.

**Fig. 1.**
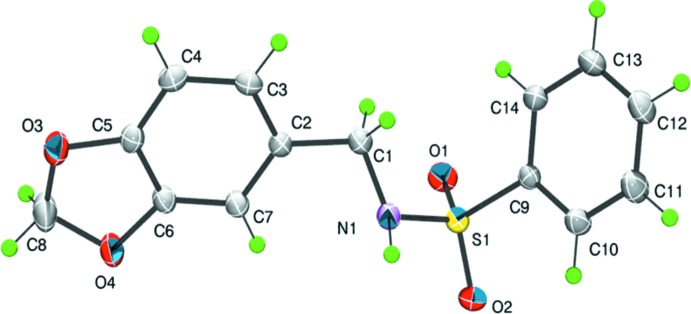
Molecular structure of the title compound showing atom labels and 50% displacement ellipsoids.

## supplementary crystallographic information

### 1. Comment

Capsaicin is the common name of *trans*-8-methyl-*N*-vanillyl-6-nonenamide, the main pungent component produced by hot red and chili peppers (Lee *et al.*, 2011). It is a product of secondary metabolism from several species of the genus *Capsicum* (Malagarie-Cazenave *et al.* 2011) The beneficial effects of capsaicinoids are well known, such as their antitumoral activity. Thus, by rational approaches, the capsaicinoid structure could be used as an important prototype for the design of novel analogues. With this in mind, we intended to design a capsaicin-like analogue with potential activity against cancer cells. The title molecule, (I), *N*-(benzo[*d*][1,3]dioxol-5-ylmethyl)benzenesulfonamide was designed by converting the vanillyl system on the capsaicinoid structure to a benzodioxol group, and modifying the alkyl-lipophilic chain by an aromatic ring. The amide bond was replaced by a sulfonamide bond using bioisosteric concepts. Molecule (I) showed relevant cytotoxicity against MCF7 breast cancer cell line presenting an IC_50_ = 32 µ*M* (De Sá-Junior *et al.*, 2013). The compound, Fig. 1, shows an envelope configuration in the 1,3-dioxole five membered ring, with the C7 atom lying 0,221 (6) Å out of the plane formed by the other four atoms. The ring puckering parameters are q2 = 0.140 (3) Å and φ2 = 145.7 (1)*^o^* (Cremer & Pople, 1975). The crystal packing of (I), Table 1, is sustained by N—H···O and C—H···π interactions, leading to supramolecular layers in the *ac* plane.

### 2. Experimental

The synthesis has been already described (De Sá-Junior *et al.*, 2013). Crystals were obtained by slow evaporation from a hexane/chloroform (4:1) solution.

### 3. Refinement

The H atoms were geometrically placed (C–H = 0.93–0.97 Å) and refined as riding with *U_iso_*(H) = 1.2*U_eq_*(C). The N—H H atom was located in a difference map and placed in that position with *U_iso_*(H) = 1.2*U_eq_*(N).

The data collection was not ideal but, in spite of that, the structure is unambiguous and fine.

### Crystal data

**Table:**

C_14_H_13_NO_4_S	*D*_x_ = 1.417 Mg m^−^^3^
*M**_r_* = 291.32	Melting point = 350.1–350.6 K
Orthorhombic, *P**b**c**a*	Mo *K*α radiation, λ = 0.71073 Å
Hall symbol: -P 2ac 2ab	Cell parameters from 3351 reflections
*a* = 18.0158 (4) Å	θ = 1.4–27.1°
*b* = 5.9346 (1) Å	µ = 0.25 mm^−^^1^
*c* = 25.5480 (8) Å	*T* = 290 K
*V* = 2731.51 (11) Å^3^	Irregular, colourless
*Z* = 8	0.25 × 0.22 × 0.20 mm
*F*(000) = 1216	

### Data collection

**Table:**

Nonius KappaCCD with Bruker APEXII CCD area detector diffractometer	2652 independent reflections
Radiation source: fine-focus sealed tube	1860 reflections with *I* > 2σ(*I*)
Graphite monochromator	*R*_int_ = 0.000
φ and ω scans	θ_max_ = 26.0°, θ_min_ = 1.6°
Absorption correction: multi-scan (*SADABS*; Sheldrick, 1996)	*h* = 0→22
*T*_min_ = 0.930, *T*_max_ = 0.948	*k* = 0→7
2652 measured reflections	*l* = −31→0

### Refinement

**Table:**

Refinement on *F*^2^	Primary atom site location: structure-invariant direct methods
Least-squares matrix: full	Secondary atom site location: difference Fourier map
*R*[*F*^2^ > 2σ(*F*^2^)] = 0.051	Hydrogen site location: inferred from neighbouring sites
*wR*(*F*^2^) = 0.149	H-atom parameters constrained
*S* = 1.04	*w* = 1/[σ^2^(*F*_o_^2^) + (0.0843*P*)^2^ + 0.6245*P*] where *P* = (*F*_o_^2^ + 2*F*_c_^2^)/3
2652 reflections	(Δ/σ)_max_ < 0.001
181 parameters	Δρ_max_ = 0.26 e Å^−^^3^
0 restraints	Δρ_min_ = −0.29 e Å^−^^3^

### Special details

**Table:**

Geometry. All s.u.'s (except the s.u. in the dihedral angle between two l.s. planes) are estimated using the full covariance matrix. The cell s.u.'s are taken into account individually in the estimation of s.u.'s in distances, angles and torsion angles; correlations between s.u.'s in cell parameters are only used when they are defined by crystal symmetry. An approximate (isotropic) treatment of cell s.u.'s is used for estimating s.u.'s involving l.s. planes.
Refinement. Refinement of *F*^2^ against ALL reflections. The weighted *R*-factor *wR* and goodness of fit *S* are based on *F*^2^, conventional *R*-factors *R* are based on *F*, with *F* set to zero for negative *F*^2^. The threshold expression of *F*^2^ > 2σ(*F*^2^) is used only for calculating *R*-factors(gt) *etc*. and is not relevant to the choice of reflections for refinement. *R*-factors based on *F*^2^ are statistically about twice as large as those based on *F*, and *R*- factors based on ALL data will be even larger.

### Fractional atomic coordinates and isotropic or equivalent isotropic displacement parameters (Å^2^)

**Table:**

	*x*	*y*	*z*	*U*_iso_*/*U*_eq_	
C1	0.14422 (14)	0.0794 (4)	0.26877 (9)	0.0610 (6)	
H1A	0.1939	0.1190	0.2574	0.073*	
H1B	0.1283	−0.0507	0.2487	0.073*	
C2	0.14588 (11)	0.0187 (4)	0.32636 (9)	0.0493 (5)	
C3	0.17158 (12)	−0.1926 (4)	0.34057 (10)	0.0561 (6)	
H3	0.1836	−0.2958	0.3145	0.067*	
C4	0.17986 (13)	−0.2546 (4)	0.39256 (10)	0.0596 (6)	
H4	0.1975	−0.3962	0.4019	0.071*	
C5	0.16096 (13)	−0.0985 (4)	0.42922 (9)	0.0550 (6)	
C6	0.13417 (14)	0.1100 (4)	0.41568 (9)	0.0559 (6)	
C7	0.12552 (13)	0.1742 (4)	0.36469 (8)	0.0547 (6)	
H7	0.1069	0.3154	0.3559	0.066*	
C8	0.1485 (2)	0.1023 (6)	0.50195 (12)	0.0966 (11)	
H8A	0.1936	0.1721	0.5148	0.116*	
H8B	0.1132	0.0948	0.5306	0.116*	
C9	0.09760 (11)	0.2425 (4)	0.15195 (8)	0.0492 (5)	
C10	0.02961 (13)	0.2300 (4)	0.12712 (10)	0.0625 (6)	
H10	−0.0093	0.3224	0.1377	0.075*	
C11	0.01987 (15)	0.0799 (5)	0.08658 (11)	0.0748 (8)	
H11	−0.0258	0.0719	0.0697	0.090*	
C12	0.07628 (17)	−0.0566 (5)	0.07097 (11)	0.0805 (8)	
H12	0.0689	−0.1577	0.0436	0.097*	
C13	0.14451 (16)	−0.0459 (5)	0.09547 (11)	0.0806 (9)	
H13	0.1830	−0.1390	0.0845	0.097*	
C14	0.15560 (13)	0.1045 (5)	0.13662 (10)	0.0647 (7)	
H14	0.2013	0.1120	0.1535	0.078*	
O1	0.18306 (9)	0.4975 (3)	0.20769 (7)	0.0703 (5)	
O2	0.04895 (10)	0.5823 (3)	0.20637 (6)	0.0654 (5)	
O3	0.16399 (11)	−0.1167 (3)	0.48341 (7)	0.0764 (6)	
O4	0.11841 (13)	0.2327 (3)	0.46019 (6)	0.0841 (6)	
N1	0.09406 (10)	0.2681 (3)	0.25825 (7)	0.0558 (5)	
H1N	0.0405	0.2323	0.2630	0.067*	
S1	0.10836 (3)	0.42069 (10)	0.20672 (2)	0.0518 (2)	

### Atomic displacement parameters (Å^2^)

**Table:**

	*U*^11^	*U*^22^	*U*^33^	*U*^12^	*U*^13^	*U*^23^
C1	0.0711 (16)	0.0610 (14)	0.0509 (14)	0.0106 (12)	0.0004 (11)	−0.0038 (11)
C2	0.0490 (12)	0.0500 (13)	0.0487 (13)	−0.0012 (10)	0.0018 (9)	−0.0018 (10)
C3	0.0558 (13)	0.0492 (14)	0.0632 (15)	0.0037 (10)	0.0044 (11)	−0.0060 (11)
C4	0.0576 (14)	0.0483 (12)	0.0728 (17)	0.0033 (11)	−0.0017 (12)	0.0047 (11)
C5	0.0579 (13)	0.0551 (13)	0.0519 (13)	−0.0040 (10)	−0.0008 (10)	0.0085 (11)
C6	0.0698 (15)	0.0511 (13)	0.0467 (13)	0.0008 (11)	0.0029 (11)	−0.0035 (10)
C7	0.0679 (14)	0.0463 (12)	0.0497 (13)	0.0031 (11)	0.0018 (11)	0.0017 (10)
C8	0.156 (3)	0.084 (2)	0.0500 (16)	0.010 (2)	−0.0048 (18)	0.0069 (15)
C9	0.0487 (12)	0.0559 (12)	0.0431 (12)	0.0012 (10)	0.0049 (9)	0.0026 (9)
C10	0.0515 (13)	0.0768 (16)	0.0592 (14)	0.0030 (12)	0.0026 (11)	−0.0098 (12)
C11	0.0678 (17)	0.0909 (19)	0.0657 (17)	−0.0066 (14)	−0.0029 (13)	−0.0167 (15)
C12	0.099 (2)	0.0810 (18)	0.0619 (17)	0.0034 (17)	−0.0010 (15)	−0.0185 (14)
C13	0.091 (2)	0.089 (2)	0.0620 (17)	0.0302 (17)	0.0082 (15)	−0.0123 (15)
C14	0.0592 (14)	0.0821 (17)	0.0526 (14)	0.0156 (12)	−0.0001 (11)	−0.0023 (12)
O1	0.0522 (10)	0.0763 (12)	0.0823 (13)	−0.0131 (9)	0.0035 (8)	−0.0066 (9)
O2	0.0653 (11)	0.0623 (11)	0.0686 (11)	0.0126 (8)	0.0045 (8)	−0.0066 (8)
O3	0.1040 (14)	0.0704 (12)	0.0548 (11)	0.0079 (10)	−0.0014 (9)	0.0142 (8)
O4	0.1363 (18)	0.0690 (12)	0.0471 (10)	0.0244 (11)	−0.0015 (10)	−0.0023 (9)
N1	0.0522 (11)	0.0670 (12)	0.0482 (11)	0.0010 (9)	0.0026 (8)	0.0001 (9)
S1	0.0522 (4)	0.0525 (4)	0.0507 (4)	−0.0022 (2)	0.0016 (2)	−0.0017 (2)

### Geometric parameters (Å, º)

**Table:**

C1—N1	1.464 (3)	C8—H8B	0.9700
C1—C2	1.515 (3)	C9—C10	1.382 (3)
C1—H1A	0.9700	C9—C14	1.384 (3)
C1—H1B	0.9700	C9—S1	1.765 (2)
C2—C3	1.385 (3)	C10—C11	1.377 (3)
C2—C7	1.395 (3)	C10—H10	0.9300
C3—C4	1.386 (3)	C11—C12	1.360 (4)
C3—H3	0.9300	C11—H11	0.9300
C4—C5	1.361 (3)	C12—C13	1.381 (4)
C4—H4	0.9300	C12—H12	0.9300
C5—C6	1.372 (3)	C13—C14	1.393 (4)
C5—O3	1.390 (3)	C13—H13	0.9300
C6—C7	1.366 (3)	C14—H14	0.9300
C6—O4	1.380 (3)	O1—S1	1.4210 (17)
C7—H7	0.9300	O2—S1	1.4372 (18)
C8—O3	1.411 (4)	N1—S1	1.6186 (19)
C8—O4	1.425 (3)	N1—H1N	0.9947
C8—H8A	0.9700		
			
N1—C1—C2	111.85 (18)	C10—C9—C14	120.5 (2)
N1—C1—H1A	109.2	C10—C9—S1	119.57 (17)
C2—C1—H1A	109.2	C14—C9—S1	119.74 (18)
N1—C1—H1B	109.2	C11—C10—C9	119.5 (2)
C2—C1—H1B	109.2	C11—C10—H10	120.2
H1A—C1—H1B	107.9	C9—C10—H10	120.2
C3—C2—C7	120.2 (2)	C12—C11—C10	120.7 (2)
C3—C2—C1	118.4 (2)	C12—C11—H11	119.6
C7—C2—C1	121.3 (2)	C10—C11—H11	119.6
C2—C3—C4	121.8 (2)	C11—C12—C13	120.3 (3)
C2—C3—H3	119.1	C11—C12—H12	119.8
C4—C3—H3	119.1	C13—C12—H12	119.8
C5—C4—C3	116.9 (2)	C12—C13—C14	119.9 (2)
C5—C4—H4	121.6	C12—C13—H13	120.0
C3—C4—H4	121.6	C14—C13—H13	120.0
C4—C5—C6	121.9 (2)	C9—C14—C13	119.0 (2)
C4—C5—O3	128.5 (2)	C9—C14—H14	120.5
C6—C5—O3	109.6 (2)	C13—C14—H14	120.5
C7—C6—C5	122.1 (2)	C5—O3—C8	104.8 (2)
C7—C6—O4	128.0 (2)	C6—O4—C8	104.6 (2)
C5—C6—O4	109.9 (2)	C1—N1—S1	118.62 (15)
C6—C7—C2	117.0 (2)	C1—N1—H1N	114.4
C6—C7—H7	121.5	S1—N1—H1N	111.9
C2—C7—H7	121.5	O1—S1—O2	119.43 (12)
O3—C8—O4	108.9 (2)	O1—S1—N1	108.42 (11)
O3—C8—H8A	109.9	O2—S1—N1	105.06 (10)
O4—C8—H8A	109.9	O1—S1—C9	108.06 (10)
O3—C8—H8B	109.9	O2—S1—C9	108.26 (10)
O4—C8—H8B	109.9	N1—S1—C9	106.99 (11)
H8A—C8—H8B	108.3		
			
N1—C1—C2—C3	160.6 (2)	C10—C9—C14—C13	0.5 (4)
N1—C1—C2—C7	−22.4 (3)	S1—C9—C14—C13	175.8 (2)
C7—C2—C3—C4	−1.8 (3)	C12—C13—C14—C9	−0.5 (4)
C1—C2—C3—C4	175.2 (2)	C4—C5—O3—C8	171.9 (3)
C2—C3—C4—C5	0.5 (3)	C6—C5—O3—C8	−8.6 (3)
C3—C4—C5—C6	0.7 (4)	O4—C8—O3—C5	14.5 (3)
C3—C4—C5—O3	−179.9 (2)	C7—C6—O4—C8	−171.0 (3)
C4—C5—C6—C7	−0.6 (4)	C5—C6—O4—C8	9.3 (3)
O3—C5—C6—C7	179.8 (2)	O3—C8—O4—C6	−14.8 (4)
C4—C5—C6—O4	179.0 (2)	C2—C1—N1—S1	155.52 (16)
O3—C5—C6—O4	−0.5 (3)	C1—N1—S1—O1	−53.6 (2)
C5—C6—C7—C2	−0.6 (4)	C1—N1—S1—O2	177.62 (16)
O4—C6—C7—C2	179.8 (2)	C1—N1—S1—C9	62.69 (19)
C3—C2—C7—C6	1.8 (3)	C10—C9—S1—O1	−149.4 (2)
C1—C2—C7—C6	−175.1 (2)	C14—C9—S1—O1	35.3 (2)
C14—C9—C10—C11	−0.4 (4)	C10—C9—S1—O2	−18.7 (2)
S1—C9—C10—C11	−175.7 (2)	C14—C9—S1—O2	165.94 (19)
C9—C10—C11—C12	0.3 (4)	C10—C9—S1—N1	94.0 (2)
C10—C11—C12—C13	−0.3 (5)	C14—C9—S1—N1	−81.3 (2)
C11—C12—C13—C14	0.4 (5)		

### Hydrogen-bond geometry (Å, º)

Cg1 and Cg2 are the centroids of the C2–C7 and C9–C14 rings, respectively.

**Table:**

*D*—H···*A*	*D*—H	H···*A*	*D*···*A*	*D*—H···*A*
N1—H1*N*···O2^i^	0.99	2.00	2.945 (3)	157
C14—H14···O1^ii^	0.93	2.59	3.486 (3)	161
C10—H10···*Cg*1^iii^	0.93	2.74	3.563 (3)	147
C8—H8*B*···*Cg*2^iv^	0.97	2.82	3.511 (3)	129

Symmetry codes: (i) −*x*, *y*−1/2, −*z*+1/2; (ii) −*x*+1/2, *y*−1/2, *z*; (iii) −*x*, *y*+1/2, −*z*+1/2; (iv) *x*, −*y*−1/2, *z*−1/2.
